# Ethyl 4-(4-nitro­phen­yl)-2-(trifluoro­meth­yl)pyrimido[1,2-*a*]benzimidazole-3-carboxyl­ate

**DOI:** 10.1107/S160053680803941X

**Published:** 2008-11-29

**Authors:** Feng-Ling Yang, Gong-Chun Li, Chang-Sheng Yao

**Affiliations:** a College of Chemistry and Chemical Engineering, Xuchang University, Xuchang, Henan Province 461000, People’s Republic of China; b School of Chemistry and Chemical Engineering and Key Laboratory of Biotechnology for Medicinal Plants, Xuzhou Normal University, Xuzhou 221116, People’s Republic of China

## Abstract

In the title compound, C_20_H_13_F_3_N_4_O_4_, the fused pyrimido[1,2-*a*]benzimidazole ring system is nearly planar, with a maximum deviation from the mean plane of 0.126 (1) Å. Mol­ecules are linked by C—H⋯N and C—H⋯O hydrogen bonds and by π–π inter­actions with inter­planar distances of 3.2661 (6) and 3.2775 (6) Å.

## Related literature

For the bioactivity of benzo[4,5] imidazo[1,2-*a*]-pyrimidine derivatives, see: Abdel-Hafez (2007 ▶); Cheung *et al.* (2002 ▶); Nunes, Zhu, Amouzegh *et al.* (2005 ▶); Nunes, Zhu, Ermann *et al.* (2005 ▶ ▶). For the bioactivity of organofluorine compounds, see: Hermann *et al.* (2003 ▶); Ulrich (2004 ▶).
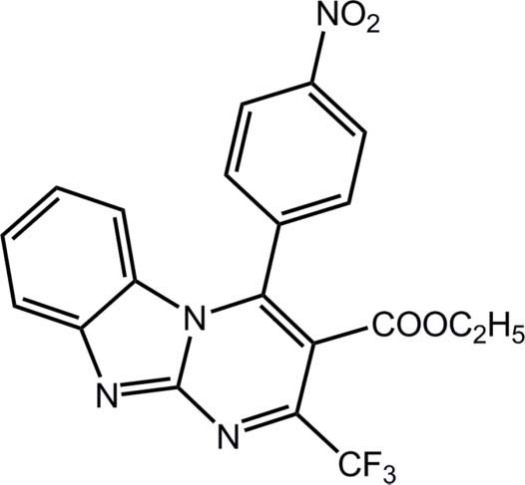


## Experimental

### 

#### Crystal data

C_20_H_13_F_3_N_4_O_4_
*M* *_r_* = 430.34
Monoclinic, 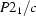

*a* = 8.4075 (5) Å
*b* = 26.6904 (14) Å
*c* = 9.0559 (5) Å
β = 111.027 (2)°
*V* = 1896.82 (18) Å^3^
*Z* = 4
Mo *K*α radiation
μ = 0.13 mm^−1^
*T* = 113 (2) K
0.32 × 0.30 × 0.26 mm

#### Data collection

Rigaku Saturn diffractometer
Absorption correction: multi-scan (*CrystalClear*; Rigaku/MSC, 2002 ▶) *T*_min_ = 0.961, *T*_max_ = 0.968
18499 measured reflections
4492 independent reflections
3911 reflections with *I* > 2σ(*I*)
*R*_int_ = 0.039

#### Refinement

*R*[*F* ^2^ > 2σ(*F* ^2^)] = 0.045
*wR*(*F* ^2^) = 0.116
*S* = 1.11
4492 reflections
282 parameters
H-atom parameters constrained
Δρ_max_ = 0.34 e Å^−3^
Δρ_min_ = −0.22 e Å^−3^

### 

Data collection: *CrystalClear* (Rigaku/MSC, 2002 ▶); cell refinement: *CrystalClear*; data reduction: *CrystalClear*; program(s) used to solve structure: *SHELXS97* (Sheldrick, 2008 ▶); program(s) used to refine structure: *SHELXL97* (Sheldrick, 2008 ▶); molecular graphics: *SHELXTL* (Sheldrick, 2008 ▶); software used to prepare material for publication: *SHELXTL*.

## Supplementary Material

Crystal structure: contains datablocks I, global. DOI: 10.1107/S160053680803941X/fj2169sup1.cif

Structure factors: contains datablocks I. DOI: 10.1107/S160053680803941X/fj2169Isup2.hkl

Additional supplementary materials:  crystallographic information; 3D view; checkCIF report

## Figures and Tables

**Table 1:** Hydrogen-bond geometry (Å, °)

*D*—H⋯*A*	*D*—H	H⋯*A*	*D*⋯*A*	*D*—H⋯*A*
C12—H12⋯N3^i^	0.95	2.41	3.2987 (18)	156
C16—H16⋯O3^ii^	0.95	2.55	3.2096 (18)	127

Symmetry codes: (i) 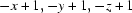; (ii) 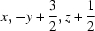.

**Fig. 1.**
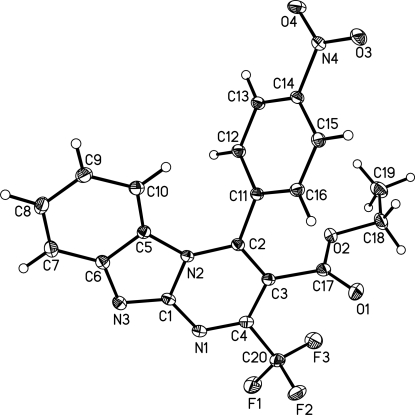
The molecular structure of the title compound, showing 30% probability displacement ellipsoids and the atom-numbering scheme.

**Fig. 2.**
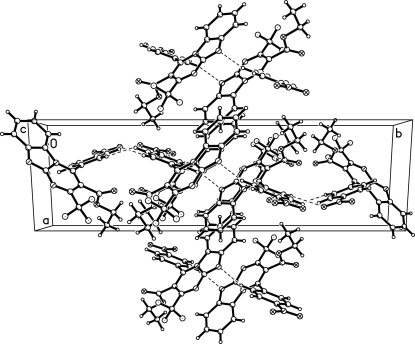
The packing diagram of the title compound. Intermolecular hydrogen bonds are shown as dashed lines.

## supplementary crystallographic information

### Comment

Among the derivatives of the dihydropyrimidine, the derivatives of pyrimido[1,2-*a*]benzimidazole have been reported to have a variety of biological activities, such as antineoplastic activity (Abdel-Hafez, 2007), protein kinase inhibitor (Nunes, Zhu, Amouzegh *et al.*, 2005), T cell activation (Nunes, Zhu, Ermann *et al.*, 2005), TIE-2 and/or VEGFR2 inhibitory activities (Cheung *et al*, 2002). Besides, compounds that contain fluorine have special bioactivity, for example, flumioxazin is a widely used herbicide (Hermann *et al.*,  2003; Ulrich,2004). This led us to pay much attention to the synthesis and bioactivity of these important fused perfluoroalkylated heterocyclic compounds. To further study the relationship between the structure and bioactivity, we synthesised series of derivatives of benzo[4,5] pyrimido[1,2-*a*]benzimidazole. Here we report the crystal structure of the title compound, (I).

In the title molecule (Fig.1), the fused ring are near planar, for the dihedral angle between the phenyl ring/imidazole ring/pyrimidine ring are 3.68 (9) and 3.65 (8)°, respectively. The conformation of the attachment of the phenyl ring to the fused ring is described by the torsion angle of N2-C2-C11-C16 of 123.17 (14)°.

The crystal packing is stabilized by C—H···N and C—H···O intermolecular hydrogen bond (Table 1, Fig. 2). In addition, there are the intermolecular π–π stacking interacions between the two neighbouring parallel imidazole rings(symmetry code: 1-x,1-y,1-z; centroid-to-centroid distance: 3.3386 (9)Å, plane-plane distance: 3.2661 (6)Å, displacement distance: 0.692Å) and phenyl rings (C5-C10, symmetry code: -x,1-y,1-z; centroid-to-centroid distance: 3.9822 (9)Å, plane-plane distance: 3.2775 (6)Å, displacement distance: 2.262Å) in the title compound.

### Experimental

The title compound was synthesized by the reaction of 4-nitrobenzaldehyde (1 mmol), ethyl 4,4,4-trifluoro-3-oxobutanoate (1 mmol) and 1*H*-benzo[*d*]imidazol-2-amine (1 mmol) in 3-butyl-1-methyl-1H- imidazol-3-ium chloride (1.5 mL) at 363 K for a certain time (monitered by TLC). After cooling, the reaction mixture was washed with water and recrystallized from ethanol, to obtain single crystals suitable for X-ray diffraction.

### Refinement

H atoms were placed in calculated positions (C-H = 0.95–0.99 Å) and included in the final cycles of refinement using a riding model, with *U*_iso_(H) = 1.2*U*_eq_(parent atom).

### Crystal data

**Table:**

C_20_H_13_F_3_N_4_O_4_	*F*_000_ = 880
*M**_r_* = 430.34	*D*_x_ = 1.507 Mg m^−^^3^
Monoclinic, *P*2_1_/*c*	Mo *K*α radiation λ = 0.71073 Å
Hall symbol: -P 2ybc	Cell parameters from 4213 reflections
*a* = 8.4075 (5) Å	θ = 2.3–27.9º
*b* = 26.6904 (14) Å	µ = 0.13 mm^−^^1^
*c* = 9.0559 (5) Å	*T* = 113 (2) K
β = 111.027 (2)º	Block, orange
*V* = 1896.82 (18) Å^3^	0.32 × 0.30 × 0.26 mm
*Z* = 4	

### Data collection

**Table:**

Rigaku Saturn diffractometer	4492 independent reflections
Radiation source: rotating anode	3911 reflections with *I* > 2σ(*I*)
Monochromator: confocal	*R*_int_ = 0.039
Detector resolution: 7.31 pixels mm^-1^	θ_max_ = 27.9º
*T* = 113(2) K	θ_min_ = 2.5º
ω scans	*h* = −11→11
Absorption correction: multi-scan(CrystalClear; Rigaku/MSC, 2002)	*k* = −34→35
*T*_min_ = 0.961, *T*_max_ = 0.968	*l* = −11→11
18499 measured reflections	

### Refinement

**Table:**

Refinement on *F*^2^	Hydrogen site location: inferred from neighbouring sites
Least-squares matrix: full	H-atom parameters constrained
*R*[*F*^2^ > 2σ(*F*^2^)] = 0.045	*w* = 1/[σ^2^(*F*_o_^2^) + (0.0565*P*)^2^ + 0.3435*P*] where *P* = (*F*_o_^2^ + 2*F*_c_^2^)/3
*wR*(*F*^2^) = 0.116	(Δ/σ)_max_ = 0.001
*S* = 1.11	Δρ_max_ = 0.35 e Å^−^^3^
4492 reflections	Δρ_min_ = −0.22 e Å^−^^3^
282 parameters	Extinction correction: SHELXL97 (Sheldrick, 2008), Fc^*^=kFc[1+0.001xFc^2^λ^3^/sin(2θ)]^-1/4^
Primary atom site location: structure-invariant direct methods	Extinction coefficient: 0.0129 (15)
Secondary atom site location: difference Fourier map	

### Special details

**Table:**

Geometry. All esds (except the esd in the dihedral angle between two l.s. planes) are estimated using the full covariance matrix. The cell esds are taken into account individually in the estimation of esds in distances, angles and torsion angles; correlations between esds in cell parameters are only used when they are defined by crystal symmetry. An approximate (isotropic) treatment of cell esds is used for estimating esds involving l.s. planes.
Refinement. Refinement of F^2^ against ALL reflections. The weighted R-factor wR and goodness of fit S are based on F^2^, conventional R-factors R are based on F, with F set to zero for negative F^2^. The threshold expression of F^2^ > 2sigma(F^2^) is used only for calculating R-factors(gt) etc. and is not relevant to the choice of reflections for refinement. R-factors based on F^2^ are statistically about twice as large as those based on F, and R- factors based on ALL data will be even larger.

### Fractional atomic coordinates and isotropic or equivalent isotropic displacement parameters (Å^2^)

**Table:**

	*x*	*y*	*z*	*U*_iso_*/*U*_eq_	
F1	0.84705 (13)	0.60521 (4)	0.95949 (11)	0.0426 (3)	
F2	0.69893 (13)	0.67273 (4)	0.89880 (11)	0.0385 (3)	
F3	0.89019 (12)	0.65773 (4)	0.79846 (11)	0.0409 (3)	
O1	0.62940 (15)	0.71967 (4)	0.55129 (14)	0.0356 (3)	
O2	0.75651 (13)	0.66746 (4)	0.43224 (12)	0.0274 (2)	
O3	0.21549 (16)	0.71512 (4)	−0.24329 (13)	0.0346 (3)	
O4	0.26759 (14)	0.64080 (4)	−0.30905 (12)	0.0308 (3)	
N1	0.57725 (14)	0.56861 (4)	0.74106 (13)	0.0219 (3)	
N2	0.37814 (14)	0.56650 (4)	0.47093 (13)	0.0189 (2)	
N3	0.37200 (15)	0.50437 (4)	0.64198 (13)	0.0229 (3)	
N4	0.26160 (15)	0.67171 (4)	−0.21060 (14)	0.0223 (3)	
C1	0.44825 (17)	0.54571 (5)	0.62568 (15)	0.0202 (3)	
C2	0.45006 (17)	0.60793 (5)	0.43043 (15)	0.0191 (3)	
C3	0.57796 (17)	0.63165 (5)	0.54926 (16)	0.0209 (3)	
C4	0.63487 (17)	0.61029 (5)	0.70372 (15)	0.0215 (3)	
C5	0.24130 (17)	0.53536 (5)	0.38824 (16)	0.0204 (3)	
C6	0.24395 (18)	0.49711 (5)	0.49625 (17)	0.0220 (3)	
C7	0.12231 (19)	0.45881 (5)	0.45180 (18)	0.0266 (3)	
H7	0.1244	0.4322	0.5222	0.032*	
C8	−0.00046 (19)	0.46102 (6)	0.30259 (18)	0.0293 (3)	
H8	−0.0837	0.4353	0.2694	0.035*	
C9	−0.0057 (2)	0.50043 (6)	0.19808 (18)	0.0293 (3)	
H9	−0.0946	0.5011	0.0973	0.035*	
C10	0.11445 (18)	0.53821 (6)	0.23755 (16)	0.0255 (3)	
H10	0.1112	0.5647	0.1664	0.031*	
C11	0.39418 (17)	0.62415 (5)	0.26248 (15)	0.0200 (3)	
C12	0.41635 (18)	0.59172 (5)	0.15022 (16)	0.0223 (3)	
H12	0.4605	0.5590	0.1804	0.027*	
C13	0.37390 (18)	0.60735 (5)	−0.00523 (16)	0.0218 (3)	
H13	0.3883	0.5857	−0.0828	0.026*	
C14	0.30986 (17)	0.65537 (5)	−0.04460 (15)	0.0204 (3)	
C15	0.28857 (18)	0.68848 (5)	0.06432 (16)	0.0235 (3)	
H15	0.2457	0.7213	0.0337	0.028*	
C16	0.33160 (18)	0.67247 (5)	0.21975 (16)	0.0226 (3)	
H16	0.3184	0.6945	0.2970	0.027*	
C17	0.65622 (18)	0.67848 (5)	0.51235 (16)	0.0246 (3)	
C18	0.8334 (2)	0.71015 (6)	0.3818 (2)	0.0351 (4)	
H18A	0.8954	0.7317	0.4735	0.042*	
H18B	0.7445	0.7305	0.3032	0.042*	
C19	0.9537 (2)	0.68897 (7)	0.3101 (2)	0.0422 (4)	
H19A	1.0469	0.6716	0.3918	0.063*	
H19B	1.0003	0.7162	0.2652	0.063*	
H19C	0.8928	0.6653	0.2263	0.063*	
C20	0.76917 (19)	0.63640 (6)	0.84092 (17)	0.0284 (3)	

### Atomic displacement parameters (Å^2^)

**Table:**

	*U*^11^	*U*^22^	*U*^33^	*U*^12^	*U*^13^	*U*^23^
F1	0.0462 (6)	0.0400 (6)	0.0245 (5)	0.0004 (5)	−0.0079 (4)	0.0038 (4)
F2	0.0469 (6)	0.0361 (5)	0.0305 (5)	−0.0008 (4)	0.0115 (4)	−0.0114 (4)
F3	0.0288 (5)	0.0560 (6)	0.0329 (5)	−0.0142 (4)	0.0050 (4)	−0.0033 (4)
O1	0.0452 (7)	0.0252 (6)	0.0389 (6)	−0.0043 (5)	0.0181 (5)	−0.0062 (5)
O2	0.0309 (6)	0.0254 (5)	0.0283 (5)	−0.0041 (4)	0.0135 (5)	0.0027 (4)
O3	0.0516 (7)	0.0228 (5)	0.0276 (6)	0.0064 (5)	0.0121 (5)	0.0081 (4)
O4	0.0405 (6)	0.0326 (6)	0.0199 (5)	0.0078 (5)	0.0116 (5)	0.0013 (4)
N1	0.0222 (6)	0.0260 (6)	0.0176 (5)	0.0036 (5)	0.0073 (5)	0.0012 (5)
N2	0.0210 (6)	0.0200 (5)	0.0162 (5)	0.0018 (4)	0.0073 (4)	0.0010 (4)
N3	0.0240 (6)	0.0228 (6)	0.0238 (6)	0.0041 (5)	0.0109 (5)	0.0038 (5)
N4	0.0244 (6)	0.0225 (6)	0.0202 (6)	0.0011 (5)	0.0082 (5)	0.0039 (5)
C1	0.0220 (6)	0.0225 (6)	0.0183 (6)	0.0055 (5)	0.0097 (5)	0.0035 (5)
C2	0.0215 (6)	0.0188 (6)	0.0190 (6)	0.0026 (5)	0.0097 (5)	0.0006 (5)
C3	0.0227 (7)	0.0219 (7)	0.0182 (6)	0.0010 (5)	0.0074 (5)	0.0002 (5)
C4	0.0216 (6)	0.0247 (7)	0.0180 (6)	0.0037 (5)	0.0069 (5)	−0.0001 (5)
C5	0.0203 (6)	0.0208 (6)	0.0217 (7)	0.0003 (5)	0.0096 (5)	−0.0024 (5)
C6	0.0231 (7)	0.0210 (7)	0.0248 (7)	0.0034 (5)	0.0122 (6)	0.0003 (5)
C7	0.0299 (7)	0.0210 (7)	0.0352 (8)	0.0005 (6)	0.0193 (6)	−0.0006 (6)
C8	0.0281 (7)	0.0271 (7)	0.0365 (8)	−0.0064 (6)	0.0163 (7)	−0.0085 (6)
C9	0.0279 (8)	0.0344 (8)	0.0254 (7)	−0.0046 (6)	0.0093 (6)	−0.0053 (6)
C10	0.0262 (7)	0.0285 (7)	0.0216 (7)	−0.0021 (6)	0.0084 (6)	−0.0012 (6)
C11	0.0199 (6)	0.0219 (6)	0.0176 (6)	−0.0010 (5)	0.0062 (5)	0.0003 (5)
C12	0.0269 (7)	0.0196 (6)	0.0209 (7)	0.0032 (5)	0.0090 (6)	0.0019 (5)
C13	0.0261 (7)	0.0212 (7)	0.0198 (6)	0.0021 (5)	0.0102 (5)	0.0010 (5)
C14	0.0205 (6)	0.0232 (7)	0.0166 (6)	−0.0010 (5)	0.0057 (5)	0.0028 (5)
C15	0.0273 (7)	0.0194 (6)	0.0227 (7)	0.0028 (5)	0.0079 (6)	0.0022 (5)
C16	0.0261 (7)	0.0215 (7)	0.0198 (7)	0.0019 (5)	0.0077 (6)	−0.0008 (5)
C17	0.0248 (7)	0.0276 (7)	0.0191 (6)	−0.0024 (6)	0.0051 (6)	−0.0003 (5)
C18	0.0380 (9)	0.0335 (8)	0.0348 (9)	−0.0097 (7)	0.0142 (7)	0.0076 (7)
C19	0.0411 (10)	0.0533 (11)	0.0367 (9)	−0.0086 (8)	0.0195 (8)	0.0058 (8)
C20	0.0301 (8)	0.0311 (8)	0.0205 (7)	−0.0003 (6)	0.0047 (6)	−0.0005 (6)

### Geometric parameters (Å, °)

**Table:**

F1—C20	1.3304 (17)	C7—C8	1.376 (2)
F2—C20	1.3365 (17)	C7—H7	0.9500
F3—C20	1.3372 (18)	C8—C9	1.405 (2)
O1—C17	1.2004 (18)	C8—H8	0.9500
O2—C17	1.3277 (17)	C9—C10	1.381 (2)
O2—C18	1.4617 (17)	C9—H9	0.9500
O3—N4	1.2239 (15)	C10—H10	0.9500
O4—N4	1.2289 (15)	C11—C16	1.3941 (19)
N1—C4	1.3056 (18)	C11—C12	1.3978 (18)
N1—C1	1.3522 (18)	C12—C13	1.3865 (18)
N2—C2	1.3719 (17)	C12—H12	0.9500
N2—C5	1.3989 (17)	C13—C14	1.3864 (19)
N2—C1	1.4235 (16)	C13—H13	0.9500
N3—C1	1.3108 (18)	C14—C15	1.3830 (19)
N3—C6	1.3851 (19)	C15—C16	1.3890 (19)
N4—C14	1.4755 (16)	C15—H15	0.9500
C2—C3	1.3729 (19)	C16—H16	0.9500
C2—C11	1.4861 (18)	C18—C19	1.495 (2)
C3—C4	1.4248 (18)	C18—H18A	0.9900
C3—C17	1.5049 (19)	C18—H18B	0.9900
C4—C20	1.516 (2)	C19—H19A	0.9800
C5—C10	1.4004 (19)	C19—H19B	0.9800
C5—C6	1.4084 (19)	C19—H19C	0.9800
C6—C7	1.399 (2)		
			
C17—O2—C18	115.89 (12)	C16—C11—C12	120.43 (12)
C4—N1—C1	117.02 (12)	C16—C11—C2	120.19 (12)
C2—N2—C5	133.83 (11)	C12—C11—C2	119.22 (12)
C2—N2—C1	120.72 (11)	C13—C12—C11	119.95 (13)
C5—N2—C1	105.42 (11)	C13—C12—H12	120.0
C1—N3—C6	104.76 (11)	C11—C12—H12	120.0
O3—N4—O4	123.49 (12)	C14—C13—C12	118.30 (12)
O3—N4—C14	118.27 (11)	C14—C13—H13	120.9
O4—N4—C14	118.23 (11)	C12—C13—H13	120.9
N3—C1—N1	125.63 (12)	C15—C14—C13	123.03 (12)
N3—C1—N2	113.11 (12)	C15—C14—N4	118.63 (12)
N1—C1—N2	121.23 (12)	C13—C14—N4	118.34 (12)
N2—C2—C3	117.29 (12)	C14—C15—C16	118.21 (13)
N2—C2—C11	120.05 (12)	C14—C15—H15	120.9
C3—C2—C11	122.61 (12)	C16—C15—H15	120.9
C2—C3—C4	118.54 (13)	C15—C16—C11	120.07 (13)
C2—C3—C17	119.40 (12)	C15—C16—H16	120.0
C4—C3—C17	122.05 (12)	C11—C16—H16	120.0
N1—C4—C3	124.75 (13)	O1—C17—O2	125.91 (14)
N1—C4—C20	114.58 (12)	O1—C17—C3	123.53 (13)
C3—C4—C20	120.66 (13)	O2—C17—C3	110.57 (12)
N2—C5—C10	132.99 (13)	O2—C18—C19	106.55 (13)
N2—C5—C6	104.70 (12)	O2—C18—H18A	110.4
C10—C5—C6	122.20 (13)	C19—C18—H18A	110.4
N3—C6—C7	127.89 (13)	O2—C18—H18B	110.4
N3—C6—C5	111.94 (12)	C19—C18—H18B	110.4
C7—C6—C5	120.13 (13)	H18A—C18—H18B	108.6
C8—C7—C6	117.67 (13)	C18—C19—H19A	109.5
C8—C7—H7	121.2	C18—C19—H19B	109.5
C6—C7—H7	121.2	H19A—C19—H19B	109.5
C7—C8—C9	121.65 (14)	C18—C19—H19C	109.5
C7—C8—H8	119.2	H19A—C19—H19C	109.5
C9—C8—H8	119.2	H19B—C19—H19C	109.5
C10—C9—C8	121.95 (14)	F1—C20—F2	107.24 (12)
C10—C9—H9	119.0	F1—C20—F3	107.08 (12)
C8—C9—H9	119.0	F2—C20—F3	107.02 (12)
C9—C10—C5	116.31 (13)	F1—C20—C4	112.34 (12)
C9—C10—H10	121.8	F2—C20—C4	110.55 (12)
C5—C10—H10	121.8	F3—C20—C4	112.33 (12)
			
C6—N3—C1—N1	176.80 (12)	C7—C8—C9—C10	1.9 (2)
C6—N3—C1—N2	−1.36 (15)	C8—C9—C10—C5	−0.5 (2)
C4—N1—C1—N3	−178.44 (13)	N2—C5—C10—C9	−177.67 (14)
C4—N1—C1—N2	−0.42 (18)	C6—C5—C10—C9	−2.0 (2)
C2—N2—C1—N3	−175.64 (11)	N2—C2—C11—C16	123.17 (14)
C5—N2—C1—N3	2.61 (15)	C3—C2—C11—C16	−59.60 (18)
C2—N2—C1—N1	6.11 (18)	N2—C2—C11—C12	−61.32 (17)
C5—N2—C1—N1	−175.64 (11)	C3—C2—C11—C12	115.92 (15)
C5—N2—C2—C3	174.80 (13)	C16—C11—C12—C13	−0.8 (2)
C1—N2—C2—C3	−7.54 (18)	C2—C11—C12—C13	−176.33 (12)
C5—N2—C2—C11	−7.8 (2)	C11—C12—C13—C14	0.0 (2)
C1—N2—C2—C11	169.83 (11)	C12—C13—C14—C15	0.9 (2)
N2—C2—C3—C4	3.79 (18)	C12—C13—C14—N4	−178.87 (12)
C11—C2—C3—C4	−173.52 (12)	O3—N4—C14—C15	4.90 (19)
N2—C2—C3—C17	−177.54 (11)	O4—N4—C14—C15	−174.11 (13)
C11—C2—C3—C17	5.16 (19)	O3—N4—C14—C13	−175.37 (13)
C1—N1—C4—C3	−3.5 (2)	O4—N4—C14—C13	5.63 (18)
C1—N1—C4—C20	175.22 (12)	C13—C14—C15—C16	−0.8 (2)
C2—C3—C4—N1	1.9 (2)	N4—C14—C15—C16	178.91 (12)
C17—C3—C4—N1	−176.76 (13)	C14—C15—C16—C11	−0.1 (2)
C2—C3—C4—C20	−176.79 (12)	C12—C11—C16—C15	0.9 (2)
C17—C3—C4—C20	4.6 (2)	C2—C11—C16—C15	176.33 (13)
C2—N2—C5—C10	−8.5 (2)	C18—O2—C17—O1	−3.0 (2)
C1—N2—C5—C10	173.60 (14)	C18—O2—C17—C3	176.91 (12)
C2—N2—C5—C6	175.31 (13)	C2—C3—C17—O1	106.37 (17)
C1—N2—C5—C6	−2.60 (13)	C4—C3—C17—O1	−75.00 (19)
C1—N3—C6—C7	−178.26 (13)	C2—C3—C17—O2	−73.58 (16)
C1—N3—C6—C5	−0.43 (15)	C4—C3—C17—O2	105.05 (14)
N2—C5—C6—N3	1.99 (14)	C17—O2—C18—C19	174.18 (13)
C10—C5—C6—N3	−174.73 (12)	N1—C4—C20—F1	22.08 (18)
N2—C5—C6—C7	−179.99 (12)	C3—C4—C20—F1	−159.12 (13)
C10—C5—C6—C7	3.3 (2)	N1—C4—C20—F2	−97.67 (14)
N3—C6—C7—C8	175.79 (13)	C3—C4—C20—F2	81.13 (16)
C5—C6—C7—C8	−1.88 (19)	N1—C4—C20—F3	142.87 (13)
C6—C7—C8—C9	−0.6 (2)	C3—C4—C20—F3	−38.33 (18)

### Hydrogen-bond geometry (Å, °)

**Table:**

*D*—H···*A*	*D*—H	H···*A*	*D*···*A*	*D*—H···*A*
C12—H12···N3^i^	0.95	2.41	3.2987 (18)	156
C16—H16···O3^ii^	0.95	2.55	3.2096 (18)	127

Symmetry codes: (i) −*x*+1, −*y*+1, −*z*+1; (ii) *x*, −*y*+3/2, *z*+1/2.
